# Postbiotics enhance immune competence in black rockfish (*Sebastes schlegelii*): linking innate immunity, apoptosis, and resistance to bacterial infection

**DOI:** 10.1186/s40104-026-01502-y

**Published:** 2026-10-04

**Authors:** Su-Jeong Lee, Young-Sun Lee, Yu-Ri Kim, Soo-Bin Hwang, Mi-Hyeon Jeon, Seong-Mok Jeong, Jinho Bae, Min-Gi Kim, Byunghwa Min, Eun-Woo Lee, Won Je Jang

**Affiliations:** 1 Department of Biomedicine and Pharmaceutics, Dong-Eui University, Busan, 47340 Korea; 2 Aquafeed Research Center, National Institute of Fisheries Science, Pohang, 37517 Korea; 3 South Sea Fisheries Research Institute, National Institute of Fisheries Science, Yeosu, 59780 Korea; 4 Research Institute for Microbiology, Dong-Eui University, Busan, 47340 Korea

**Keywords:** Apoptosis, Disease resistance, Immune modulation, Microbiota, Postbiotics

## Abstract

**Background:**

This study investigated the functional potential and safety of postbiotics derived from *Latilactobacillus curvatus* NBL2305 as a dietary feed additive for juvenile black rockfish (*Sebastes schlegelii*). Fish were fed diets supplemented with NBL2305-derived postbiotics, and their effects on growth performance, immune responses, antioxidant capacity, apoptosis-related gene expression, intestinal microbiota, and disease resistance were evaluated.

**Results:**

Supplementation with postbiotics significantly improved growth performance and feed utilization, as indicated by increased weight gain and specific growth rate, along with a reduced feed conversion ratio. Innate immune responses were enhanced, as demonstrated by elevated activities of myeloperoxidase, superoxide dismutase, and lysozyme together with the upregulated expression of immune-related genes, including *il8*, *il1b*, *tnfa*, *tnfsf13b*, and *nfkb1*. In addition, postbiotic treatment modulated apoptosis-related gene expression in the liver, characterized by the downregulation of pro-apoptotic genes (*bax*, *tp53*, and *casp3*) and the upregulation of anti-apoptotic genes (*bcl2* and *xiap*), indicating an anti-apoptotic gene expression profile. The postbiotics also exhibited strong antimicrobial activity against major fish pathogens and considerable antioxidant capacity. These functional effects were further supported by an in vivo challenge test, in which the postbiotic-fed group showed significantly higher survival following *Edwardsiella tarda* infection. No adverse effects were observed in serum biochemical parameters, somatic indices, or histopathological analyses, supporting the short-term safety of supplementation under the experimental conditions evaluated. Moreover, the intestinal microbiota composition remained stable, with selective enrichment of beneficial taxa.

**Conclusions:**

Collectively, these findings demonstrate that NBL2305-derived postbiotics are safe and effective functional feed additives that enhance growth, modulated innate immune responses, and disease resistance in aquaculture species.

**Supplementary Information:**

The online version contains supplementary material available at https://doi.org/10.1186/s40104-026-01502-y.

## Introduction

Bacterial diseases remain a major challenge in aquaculture, often causing mass mortality in fish and posing a serious threat to industry sustainability [1]. The widespread and indiscriminate use of antibiotics for disease prevention and treatment has led to the emergence of antibiotic-resistant pathogens, reducing treatment efficacy while raising concerns regarding environmental contamination and food safety [2]. Although vaccines have been developed for several bacterial diseases in aquaculture, their protective efficacy can vary depending on pathogen diversity, fish developmental stage, and farming conditions [3, 4]. In addition, vaccines are not available for all bacterial pathogens and may be less effective under certain production conditions. Therefore, there is an urgent need for safe and sustainable alternatives to antibiotics for disease control in aquaculture systems.

Microbial feed additives, particularly probiotics, have gained considerable attention as environmentally friendly strategies for improving fish health, controlling bacterial diseases, and supporting sustainable aquaculture [5]. A wide range of probiotic species, including *Lactobacillus*, *Lactococcus*, *Leuconostoc*, *Enterococcus*, *Bacillus*, *Vibrio*, *Enterobacter*, and *Pseudomonas*, have been applied in aquaculture [6–9]. These probiotics enhance growth performance, improve feed utilization, and modulate host immune responses. However, despite their general safety, they are not entirely risk-free. Potential concerns include undesirable metabolic activities, horizontal transfer of antibiotic resistance or virulence genes, and opportunistic infections under certain host or environmental conditions [6, 10].

In response to these limitations, postbiotics have attracted increasing interest. The International Scientific Association for Probiotics and Prebiotics defines postbiotics as “a preparation of non-living microorganisms and/or their components that confer a health benefit on the host” [11]. Compared with live probiotics, postbiotics offer several advantages, including improved safety, greater stability during storage and feed processing, and elimination of risks associated with microbial translocation or infection [12]. Comprising inactivated microbial cells and their bioactive metabolites, postbiotics exhibit antimicrobial activity against fish pathogens and can inhibit intestinal pathogen colonization, thereby helping prevent major bacterial diseases such as vibriosis and photobacteriosis [13, 14]. In addition, they have been reported to improve growth performance, enhance feed efficiency, and modulate intestinal microbiota composition in aquaculture species [15, 16].

Beyond these benefits, postbiotics are increasingly recognized for their immunomodulatory effects [17]. In fish, innate immunity serves as the primary defense against invading pathogens, and modulation of non-specific immune responses, including cytokine production and antimicrobial enzyme activity, is a key mechanism underlying disease resistance [18–20]. Oxidative stress is closely linked to immune function and physiological stability, and antioxidant capacity plays a crucial role in maintaining cellular homeostasis during immune activation [21, 22]. In addition, regulation of apoptosis is essential for maintaining tissue integrity, particularly in metabolically active organs such as the liver [23]. The balance between pro-apoptotic and anti-apoptotic signaling pathways is critical for cellular homeostasis, and its disruption can lead to tissue damage and impaired physiological function [24, 25]. Although several recent studies have reported beneficial effects of postbiotics in fish, most have primarily focused on growth performance or basic innate immune responses [11, 26]. Consequently, the potential interplay among innate immunity, apoptosis-related regulation, and intestinal microbiota remains poorly understood [27, 28]. Since immune activation is closely associated with cellular homeostasis and host–microbiota interactions [29], an integrated evaluation of these biological responses is necessary to better understand the overall physiological effects of postbiotics in aquaculture species. Therefore, comprehensive studies integrating immune responses, antioxidant capacity, apoptosis, intestinal microbiota, and histopathological safety remain limited [13, 26, 30, 31].

Black rockfish (*Sebastes schlegelii*) is a commercially important marine species widely cultured in East Asia, including Korea, Japan, and China [32, 33]. Among these, *Edwardsiella tarda* is one of the major bacterial pathogens affecting marine aquaculture species, including black rockfish (*Sebastes schlegelii*), causing edwardsiellosis characterized by septicemia, high mortality, and substantial economic losses [34, 35]. The development of effective and safe microbial feed additives for this species is therefore of considerable interest. Recent studies have investigated functional feed additives in black rockfish and identified host-associated probiotic candidates from the intestinal tract [33, 36]. However, the functional characterization of these strains as postbiotic-producing candidates remains limited.

Accordingly, the present study aimed to evaluate the parental strain for its safety and probiotic-related functional properties prior to postbiotic preparation and to investigate the effects of postbiotics derived from *Latilactobacillus curvatus* NBL2305 on growth performance, immune responses, antioxidant capacity, apoptosis-related gene expression, and intestinal microbiota in juvenile black rockfish.

## Materials and methods

### Bacterial isolation and identification

Bacteria were isolated from the intestines of both wild and cultured black rockfish. Briefly, intestinal samples were homogenized and suspended in phosphate-buffered saline (PBS). The suspension was serially diluted with PBS, spread onto de Man, Rogosa, and Sharpe (MRS; MBcell, Seoul, Korea) agar plates, and incubated at 37 °C for 48 h. Individual colonies were isolated and identified by 16S ribosomal RNA gene sequencing using the 27 F and 1492R primer sets, and were stored at −80 °C in 25% glycerol until further use [37]. Biochemical identification was performed using an API^®^ 50 CHL kit (bioMérieux, France), which assesses the fermentation of 49 carbohydrates. During incubation in the test strips, carbohydrate fermentation produces acid, leading to a decrease in pH. This change is detected by a pH indicator in each cupule, allowing results to be recorded as positive or negative. All procedures were conducted according to the manufacturer’s instructions.

### Bacterial characterization

#### Postbiotics preparation

**Fig. 1:**
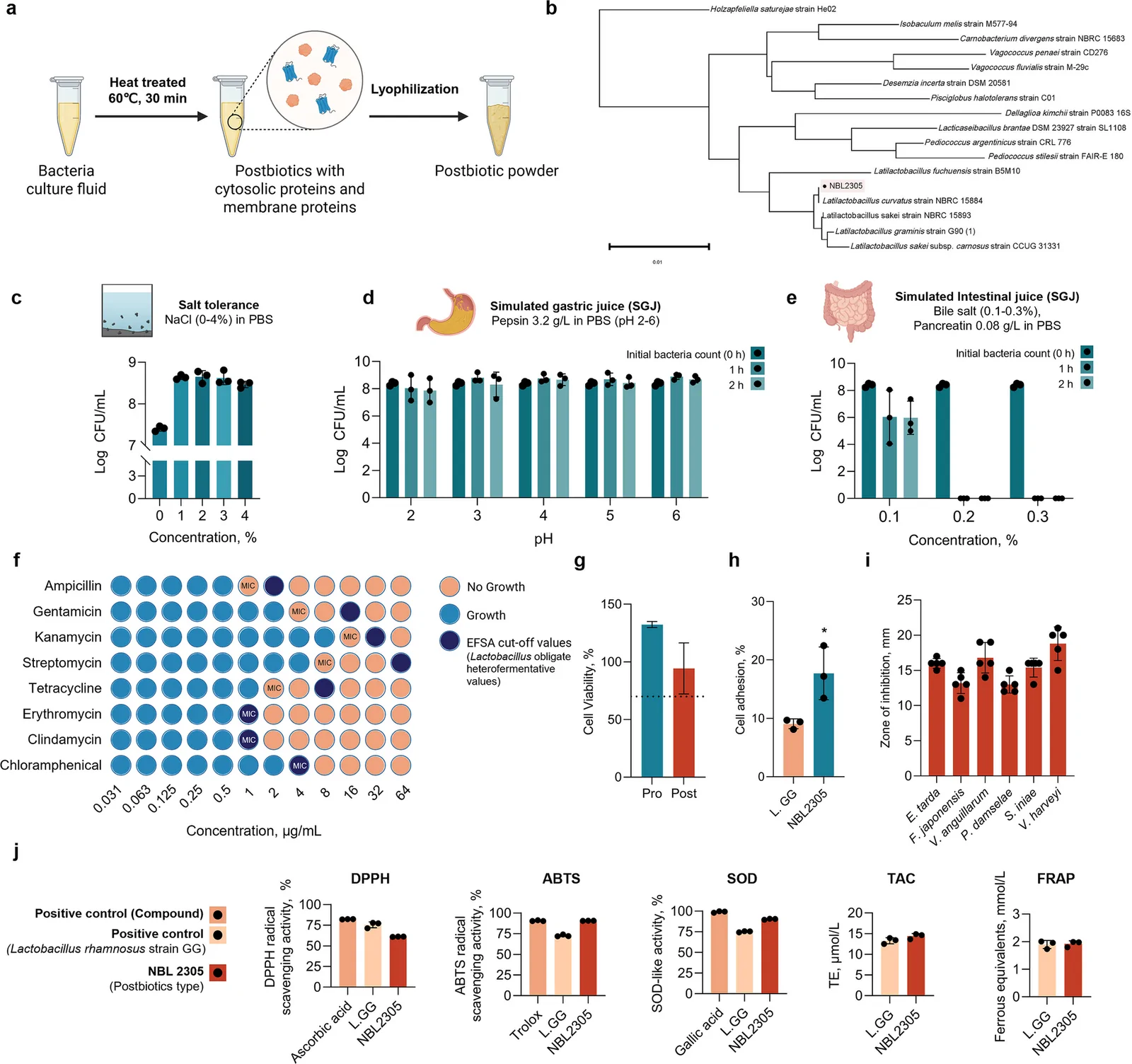
Functional characterization of *Latilactobacillus curvatus* NBL2305 as probiotics and postbiotics. **a** Schematic illustration of the preparation procedure for postbiotics derived from *L. curvatus* NBL2305. **b** Phylogenetic tree based on 16S rRNA gene sequences showing the taxonomic position of strain NBL2305 among closely related *Latilactobacillus* species. **c**–**e** Survival of the strain under salt tolerance and simulated gastrointestinal conditions, including simulated gastric and intestinal fluids. **f** Antibiotic susceptibility profile of *L. curvatus* NBL2305 against commonly used antibiotics. **g** Effects of probiotic cells and postbiotic preparations on HT-29 cell viability. **h** Adhesion ability of *L. curvatus* NBL2305 to HT-29 cells, expressed as percentage adhesion. **i** Antimicrobial activity of the postbiotic preparation against six fish pathogens, including* Edwardsiella tarda*, *Flexibacter japonensis*, *Vibrio anguillarum*, *Photobacterium damselae* subsp. *damselae*, *Streptococcus iniae*, and *Vibrio harveyi*, evaluated using an agar diffusion assay and expressed as inhibition zone diameter (mm). **j** Antioxidant activities of the postbiotic supernatant determined by DPPH, ABTS, SOD, TAC, and FRAP assays. Data are presented as mean ± SD, and statistical significance was determined using Student’s *t*-test (*P* < 0.05)

Postbiotics were prepared to include both the culture supernatant and heat-inactivated bacterial cells (Fig. 1a). NBL2305 was cultured at 37 °C for 24 h, followed by heat treatment at 60 °C for 30 min using a heating bath. Complete inactivation was confirmed by spread-plating the treated samples onto agar plates and verifying the absence of colony formation. The resulting postbiotics were freeze-dried for long-term storage and used in subsequent experiments.

#### Simulated gastrointestinal survival and salt tolerance of the parental strain

Cell viability under simulated gastrointestinal conditions was assessed following a previously described method [33]. Briefly, bacterial survival after 2 h of exposure to simulated gastric fluid (SGF) containing 3.2 g/L pepsin powder (Sigma-Aldrich, USA) and 2 g/L sodium chloride was determined by counting colony-forming units (CFU) on agar plates. To evaluate survival under simulated intestinal fluid (SIF), bacteria were incubated for 3 h in a pH 5.9 buffer containing 0.3% bile salts (Sigma-Aldrich, USA) and 0.08 g/L pancreatin, and CFU values were determined. Survival relative to the initial bacterial count was calculated using the equation: survival (%) = (*N*/*N*_0_) × 100, where *N* is the CFU after treatment and *N*_0_ is the initial CFU. Salt tolerance was evaluated to determine the ability of the strain to survive under seawater-like conditions (30 ppt salinity). NaCl was added to MRS broth at final concentrations ranging from 2% to 6% (w/v). NBL2305 was cultured at 37 °C for 24 h, and viable cell counts were measured and compared with those obtained from MRS broth without NaCl supplementation.

#### Safety evaluation

The safety of the isolated strain was assessed through hemolysis, antibiotic susceptibility, and cytotoxicity analyses. Hemolytic activity was evaluated on blood agar base plates (MBcell, Seoul, Korea) after incubation at 37 °C for 24 h. Antibiotic susceptibility was determined by measuring the minimum inhibitory concentrations (MIC) of eight antibiotics: ampicillin, gentamicin, kanamycin, streptomycin, tetracycline, erythromycin, clindamycin, and chloramphenicol. Cytotoxicity was assessed using Human colorectal adenocarcinoma cell line (HT-29) cells and a Cellrix viability assay kit (Medifab, Seoul, Korea). *Bacillus cereus* strains obtained from the Korean Collection for Type Cultures (KCTC 1012 and 3624, Korea) were used as controls in both hemolysis and cytotoxicity assays.

#### Adhesion ability of the parental strain

The adhesion ability of strain NBL2305 was evaluated using a modified method based on previously reported protocols [38]. HT-29 cells were seeded in 12-well plates at a density of 1 × 10^5^ cells/mL and incubated for 24 h. Bacterial suspensions (1 × 10^8^ CFU/mL) were then added and incubated for 2 h at 37 °C. After washing with Dulbecco's phosphate-buffered saline (DPBS) to remove non-adherent bacteria, the cells were further incubated in antibiotic-free Dulbecco’s modified Eagle’s medium (DMEM) for 24 h. Adherent bacteria were recovered by treatment with 0.25% trypsin–EDTA, and adhesion ability was calculated as the ratio of adherent bacterial CFU to the initially inoculated CFU.

#### Antibacterial activity

Six fish pathogenic bacteria (*Edwardsiella tarda*, *Flexibacter japonensis*, *Listonella anguillarum*, *Photobacterium damselae* subsp. *damselae*, *Streptococcus iniae*, and *Vibrio harveyi*) were obtained from the Korean Collection for Type Cultures (KCTC, Daejeon, Korea). All pathogens were cultured at 30 °C in Luria–Bertani (LB; Difco, USA) broth supplemented with 2% (w/v) NaCl. Antibacterial activity was evaluated using an agar well diffusion assay. Each pathogenic strain was adjusted to a 0.5 McFarland standard, and 100 μL of the suspension was evenly spread onto LB agar plates. Wells (8 mm in diameter) were punched into the agar using a sterile cork borer, and 100 μL of postbiotics was added to each well. The plates were incubated at 30 °C for 12 h, and the diameter of the clear inhibition zones was measured.

#### Antioxidant activity

Antioxidant activity was assessed using 2,2-diphenyl-1-picrylhydrazyl (DPPH), 2,2-azino-bis-3-ethylbenzothiazoline-6-sulphonic acid (ABTS), superoxide dismutase (SOD), total antioxidant capacity (TAC), and ferric reducing antioxidant power (FRAP) assays. Postbiotics were prepared as described above, followed by centrifugation and filtration through a 0.22 μm membrane filter prior to use. In all assays, antioxidant standard compounds and *Lactobacillus rhamnosus* GG were included as positive controls. For the DPPH assay, a 0.2 mmol/L DPPH solution was mixed with the sample at a 1:1 (v/v) ratio and incubated in the dark at room temperature for 30 min. Absorbance was then measured at 517 nm. For the ABTS assay, a stock solution was prepared by mixing 7.4 mmol/L ABTS with 2.45 mmol/L potassium persulfate and incubating the mixture in the dark for 16 h. The solution was diluted to an absorbance of 0.70 ± 0.05 at 734 nm, mixed with the sample, and incubated at room temperature for 6 min before measuring absorbance at 734 nm. SOD activity was determined using an EZ-SOD Assay Kit (DoGenBio, Korea) according to the manufacturer’s instructions, and results were expressed as the superoxide anion inhibition rate. TAC was measured using an EZ-TAC assay kit (DoGenBio, Republic of Korea) following the manufacturer’s protocol. Briefly, 100 μL of postbiotics was mixed with 100 μL of copper reagent and 100 μL of reaction buffer, incubated at room temperature for 30 min, and absorbance was measured at 450 nm. TAC was expressed as Trolox equivalents (TE) using a Trolox standard curve. FRAP was determined using a Ferric Reducing Antioxidant Power Assay Kit (MAK509, Sigma-Aldrich, USA) according to the manufacturer’s instructions. Briefly, samples were mixed with the FRAP working reagent, incubated at room temperature for 60 min, and absorbance was measured at 594 nm. FRAP values were calculated using a ferrous standard curve and expressed as Fe^2+^ equivalents.

### Feeding trials

#### Experimental diets

Experimental diets were formulated based on the feed formulation table (Table S1) provided by the Aquafeed Research Center of the National Institute of Fisheries Science (NIFS, Pohang, Korea). All ingredients were thoroughly mixed, extruded into pellets, and dried. The pellets were adjusted to an appropriate size for juvenile fish and stored at −20 °C until use. For the postbiotics-supplemented diet, NBL2305 was cultured under optimal conditions, freeze-dried, and incorporated into the FM35 diet at 0.3% (3 g/kg diet). The freeze-dried postbiotics were stored at −80 °C until feed preparation. The CON group was fed the control diet without postbiotic supplementation, whereas the POST group was fed the postbiotics-supplemented diet.

#### Animal and experimental condition

Juvenile black rockfish (*Sebastes schlegelii*) were obtained from the NIFS and used in an 8-week feeding trial. Fish with an initial average body weight of approximately 8 g were randomly assigned to dietary treatments at a density of 20 fish per 120 L tank, with three replicate tanks per group. Rearing conditions were maintained at 17 ± 0.3 °C, pH 7.1 ± 0.3, dissolved oxygen 6.4 ± 0.4 mg/L, and salinity 31 ± 2 ppt throughout the experimental period. Continuous aeration was provided using air stones installed in each tank, and both aeration and water flow rates were kept constant. Seawater was treated by sedimentation, pre-filtration, and ultraviolet (UV) sterilization prior to use, and partial water exchange was performed daily. Fish were fed twice daily at 10:00 and 16:00.

### Growth performance, feed utilization, and somatic indices

Growth performance, feed utilization, and somatic indices were evaluated using initial and final body weights measured after a 24-h fasting period at the end of the 8-week feeding trial. Five fish were randomly selected from each tank to determine growth parameters, including weight gain (WG, %), specific growth rate (SGR, %/d), protein efficiency ratio (PER), and feed conversion ratio (FCR), as well as hepatosomatic index (HSI), viscerosomatic index (VSI), and condition factor (CF). All parameters were calculated using the following equations:

$$\mathrm{WG} \ \left(\%\right) = [\left(\text{final body weight}-\text{initial body weight}\right) /\text{initial body weight}]\times 100$$

$$\mathrm{SGR}\ \left(\%/\mathrm{d}\right) = [\left(\text{ln final body weight}-\text{ln initial} \ \text{body weight}\right)/ \mathrm{days}] \times 100$$

$$\mathrm{PER} = \text{wet weight gain} / \text{protein intake}$$

$$\mathrm{FCR} = \text{dry feed intake} / \text{wet body weight gain}$$

$$\mathrm{HSI} \ \left(\%\right) = \left(\text{liver weight}/ \text{body weight}\right)\times 100$$

$$\mathrm{VSI}\ \left(\%\right)= \left(\text{visceral weight} / \text{body weight}\right)\times 100$$

$$\text {CF}= [\text {fish weight} \ (\text g)/ \text {fish length} \ (\text {cm})^{3}]\times100$$

### Sample collection

Following the growth measurements, five fish from each of the three replicate tanks (15 fish per dietary group) constituted the sampling pool for subsequent analyses. Blood samples were collected from five randomly selected fish per group from the caudal vein using heparinized syringes and centrifuged at 3,000 × *g* for 20 min to obtain serum, which was stored at −80 °C until analysis. The collected serum was used for the determination of serum biochemical parameters and non-specific immune responses. Following blood collection, the fish were dissected, and the liver and intestine were collected for subsequent analyses. Liver and intestinal tissues from five randomly selected fish per group were used for RT-qPCR analysis, tissues from three randomly selected fish per group were used for histological analysis, and intestinal samples from four randomly selected fish per group were used for intestinal microbiota analysis by 16S rRNA gene sequencing.

### Non-specific immune and serum biochemical parameters

Non-specific immune parameters, including SOD, lysozyme, and myeloperoxidase (MPO) activities, were measured in the serum. SOD activity was determined using an EZ-SOD Assay Kit (DoGenbio, Korea), based on the inhibition of water-soluble tetrazolium salt-1 (WST-1) reduction by superoxide radicals generated from the xanthine–xanthine oxidase system. Lysozyme activity was measured using a *Micrococcus lysodeikticus* suspension (0.2 mg/mL), where one unit was defined as a decrease in absorbance of 0.001 per minute at 530 nm. Peroxidase activity was assessed using Hanks’ Balanced Salt Solution (HBSS) and 3,3′,5,5′-tetramethylbenzidine (TMB), with absorbance recorded at 450 nm after terminating the reaction with 0.5 mol/L sulfuric acid (H_2_SO_4_). Serum biochemical parameters, including aspartate aminotransferase (AST), alanine aminotransferase (ALT), glucose (GLU), total cholesterol (TCHO), triglycerides (TG), and total protein (TP), were analyzed using a FUJI DRI-CHEM NX500i analyzer (Fujifilm, Japan).

### Analysis of digestive enzymes

Digestive enzyme activities in the anterior midgut of black rockfish were analyzed using commercial assay kits for amylase (ab102523; Abcam, UK), trypsin (ab102531; Abcam, UK), and lipase (ab102524; Abcam, UK). Five fish per group were randomly selected, and approximately 100 mg of anterior midgut tissue was collected and homogenized in PBS using a homogenizer (HZ1; LabTron, Korea). The homogenates were centrifuged at 10,000 × *g* for 10 min, and the supernatants were used for enzyme activity assays. Enzyme activities were measured colorimetrically by monitoring absorbance at 405 nm for amylase and trypsin, and at 412 nm for lipase. One unit (U) of enzyme activity was defined as the amount of enzyme required to generate 1.0 μmol of reaction product per minute under the assay conditions. All procedures were performed in accordance with the manufacturer’s protocols.

### Challenge test

The pathogenic bacterium *E. tarda* (ATCC 15947) was obtained from the Korean Collection for Type Cultures (KCTC, Korea). The strain was cultured in brain heart infusion (BHI) broth at 30 °C for 24 h, harvested by centrifugation, and washed three times with sterile PBS. The bacterial suspension was adjusted to 1 × 10^8^ CFU/mL based on viable cell counts. The bacterial concentration was selected based on preliminary challenge experiments and previous studies to induce reproducible mortality in the control group while allowing evaluation of the protective effects of dietary supplementation. At the end of the feeding trial, five juvenile black rockfish were randomly selected from each tank (*n* = 15 fish per dietary group) and anesthetized with 2-phenoxyethanol. Each fish was intraperitoneally injected with 100 μL of the bacterial suspension. Post-challenge mortality was monitored every 12 h for 14 d. Cumulative survival rates were calculated, and Kaplan–Meier survival curves were constructed. Dead fish were aseptically sampled, and tissue swabs were streaked onto BHI agar plates for bacterial re-isolation. Representative isolates were subsequently confirmed as *E. tarda* by 16S rRNA gene PCR using the primers described by Ma et al. [39].

### Gene expression analysis

Reverse transcription-quantitative polymerase chain reaction (RT-qPCR) was performed to assess the expression of growth- and immune-related genes. Liver tissues were collected and immediately processed for total RNA extraction using the Hybrid-R™ kit (GeneAll, Korea) according to the manufacturer’s instructions. The purity and concentration of the extracted RNA were determined prior to complementary DNA (cDNA) synthesis. cDNA was synthesized using the PrimeScript™ 1 st Strand cDNA Synthesis Kit (TaKaRa, Japan). Quantitative PCR amplification was performed using TB Green^®^ Premix Ex Taq (TaKaRa, Japan) on a CFX Opus 96 real-time PCR system (Bio-Rad, USA). Relative gene expression levels were calculated using the 2^−ΔΔCt^ method, with beta-actin (β-actin) used as the internal reference gene. Primer sequences used for amplification are provided in Table S2 and were designed based on previously reported sequences [40] or obtained from National center for biotechnology information (NCBI) GenBank and designed using basic local alignment search tool (BLAST).

### Histological analysis

After an 8-week feed trial, liver and intestinal tissues were collected from juvenile rockfish and subjected to histological analysis. Tissue samples were fixed in 10% neutral buffered formalin, washed under running tap water, dehydrated with stepwise ethanol solutions, cleared with xylene, and then embedded in paraffin according to the previously described method [41]. The paraffin-embedded tissues were sectioned to a thickness of 5 μm using a rotary microtome (RM2125 RTS, Leica Biosystems, Germany). The sections were stained with Hematoxylin–eosin (H&E) using a commercially available staining kit (Abcam, UK), and histological observation and image acquisition were performed using an optical microscope (BA310, Motic, China).

### Microbiome analysis

Microbial DNA was extracted from intestinal samples using the AccuPrep® Genomic DNA Extraction Kit (Bioneer, South Korea). Sequencing of the 16S rRNA gene (V3–V4 regions) was performed following the Illumina 16S metagenomic sequencing library protocol using the following primers (5′→3′): Forward primer, TCG TCG GCA GCG TCA GAT GTG TAT AAG AGA CAG CCT ACG GGN GGC WGC AG; Reverse primer (5′→3′), GTC TCG TGG GCT CGG AGA TGT GTA TAA GAG ACA GGA CTA CHV GGG TAT CTA ATC C. PCR amplification was carried out using 2 × KAPA HiFi HotStart ReadyMix (Roche, USA). PCR products were purified using magnetic beads (Agencourt AMPure XP-Kit; Beckman Coulter). Index PCR was performed using the Nextera XT Index Kit v2 Set B and 2 × KAPA HiFi HotStart ReadyMix, and the libraries were quantified using a Qubit 4 Fluorometer with the Qubit dsDNA High Sensitivity Assay Kit (Thermo Fisher Scientific, USA). The libraries were denatured with NaOH, diluted, mixed with 5% PhiX control, and sequenced on a MiSeq i100 plus platform (Illumina, USA) using a 2 × 300 cycle V3 kit. Paired-end reads (2 × 300 bp) were processed using Quantitative Insights Into Microbial Ecology 2 (QIIME2) version 2024.10 (https://qiime2.org/) [42]. Sequences were f​i​ltered, denoised, and merged, and chimeric sequences were removed using the Divisive Amplicon Denoising Algorithm 2 (DADA2) pipeline (https://github.com/benjjneb/dada2/), resulting in the generation of amplicon sequence variants (ASVs) [43]. After quality filtering and denoising, sequencing depth ranged from 100,324 to 182,031 reads per sample, with an average of 141,022 reads per sample. Prior to alpha- and beta-diversity analyses, all samples were rarefied to a uniform sequencing depth of 98,104 reads per sample. Taxonomic classification of ASVs was performed using the ‘sklearn’ classifier method with the SILVA database (version 138; https://www.arb-silva.de/). Reads that could not be taxonomically assigned were removed using the taxa filter-table function. For integrative analyses, MicrobiomeAnalyst 2.0 was used [44]. Functional prediction of the intestinal microbiota was performed using Phylogenetic investigation of communities by reconstruction of unobserved states 2 (PICRUSt2) based on the ASV abundance table and representative 16S rRNA gene sequences [45]. Predicted MetaCyc pathways were compared between the CON and POST groups. Pathways showing nominal differences (raw *P* < 0.05) were categorized according to their biological functions and are presented in Table S4.

### Statistical analysis and data visualization

Data visualization was performed using GraphPad Prism^®^ version 10 (GraphPad Software Inc., San Diego, CA, USA) and Python packages, including Seaborn and Matplotlib. All results are expressed as mean ± standard deviation (SD). Comparisons between the CON and POST groups were performed using Student's *t*-test. Cumulative survival following the *Edwardsiella tarda* challenge was analyzed using the Kaplan–Meier method, and differences between survival curves were evaluated using the log-rank (Mantel–Cox) test in GraphPad Prism. A *P*-value < 0.05 was considered statistically significant. All statistical analyses were performed using IBM SPSS Statistics version 26.0 (IBM Corp., Armonk, NY, USA).

## Results

### Taxonomic identification

Strain NBL2305, isolated from the intestine of juvenile black rockfish, was taxonomically identified based on 16S rRNA gene sequence analysis. The strain exhibited the highest sequence similarity (99.86%) to *Latilactobacillus curvatus* (NR_113334.1), supporting its assignment to this species (Fig. 1b). In addition, carbohydrate utilization profiling using the API 50 CHL kit demonstrated that NBL2305 could metabolize seven carbohydrates, including galactose (Table S3).

### Characterization

#### Tolerance to simulated gastrointestinal conditions and salinity

The tolerance of strain NBL2305 to salinity and simulated gastrointestinal conditions was evaluated. Under NaCl conditions (0–4%), viable cell counts increased from 7.41 ± 0.06 log CFU/mL at 0% NaCl to 8.47 ± 0.08 log CFU/mL at 4% NaCl, indicating that the strain maintained high viability under saline conditions (Fig. 1c). Under SGF conditions, viable cell counts at pH 2 decreased from 8.39 ± 0.14 log CFU/mL at 0 h to 7.21 ± 0.89 log CFU/mL after 2 h. At pH 4–6, viable cell counts remained above 8.0 log CFU/mL after 2 h of incubation (Fig. 1d). Under SIF conditions, at 0.1% bile salt concentration, viable cell counts decreased from 8.39 ± 0.14 log CFU/mL at 0 h to 5.48 ± 1.19 log CFU/mL at 2 h, indicating partial survival. No viable cells were detected at 0.2% and 0.3% bile salt concentrations after 1 h of exposure (Fig. 1e).

#### Safety evaluation

The safety of strain NBL2305 was assessed through hemolysis and cytotoxicity analyses. Hemolytic activity on blood agar plates showed that NBL2305 exhibited γ-hemolysis, indicating no hemolytic activity (data not shown). Antibiotic susceptibility was evaluated by determining the MIC for selected antibiotics (Fig. 1f). All MIC values for NBL2305 were below or equal to the cut-off values established by the European Food Safety Authority (EFSA), indicating susceptibility to the tested antibiotics. Cytotoxicity was assessed using a cell viability assay with the HT-29 cell line (Fig. 1g). Both the probiotic (PRO) and postbiotic (POST) forms of NBL2305 showed high cell viability, with survival rates exceeding 70% in all treatments. The Pro group showed a cell viability of 132.38% ± 2.56%, while the POST group showed 94.30% ± 22.23%. These results indicate that NBL2305 did not exhibit cytotoxic effects under the tested conditions.

#### Adhesion ability

The adhesion ability of strain NBL2305 to HT-29 cells was evaluated and compared with that of the positive control, *Lactobacillus rhamnosus* GG (Fig. 1h). The adhesion rate of NBL2305 (17.71% ± 4.50%) was significantly higher than that of *L. rhamnosus* GG (9.07% ± 0.85%) (*P* = 0.031).

#### Antibacterial activity

The antibacterial activity of NBL2305 postbiotics was confirmed against the six tested fish pathogens (Fig. 1i). The inhibition zone diameters were 16.00 ± 0.71 mm for *E. tarda*, 13.20 ± 1.48 mm for *F. japonensis*, 16.80 ± 2.17 mm for *L. anguillarum*, 13.00 ± 1.22 mm for *P. damselae*, 15.40 ± 1.34 mm for *S. iniae*, and 18.80 ± 2.39 mm for *V. harveyi*.

#### Antioxidant activity

The antioxidant activity of NBL2305 postbiotics was evaluated using DPPH and ABTS radical scavenging assays, as well as SOD activity, TAC, and FRAP assays (Fig. 1j). In the DPPH assay, NBL2305 showed a radical scavenging activity of 61.04% ± 0.26%, compared with 75.41% ± 3.62% for *L. rhamnosus* GG and 82.23% ± 0.21% for ascorbic acid. In the ABTS assay, NBL2305 exhibited a radical scavenging activity of 90.58% ± 0.13%, which was higher than that of *L. rhamnosus* GG (72.60% ± 1.18%) and comparable to that of Trolox (90.57% ± 0.48%). In the SOD assay, NBL2305 showed an activity of 90.14% ± 0.61%, whereas *L. rhamnosus* GG and gallic acid showed activities of 75.09% ± 0.73% and 98.99% ± 0.96%, respectively. In addition, the TAC and FRAP values of NBL2305 were 14.39 ± 0.64 μmol/L and 1.92 ± 0.11 mmol/L, respectively, compared with 13.29 ± 0.73 μmol/L and 1.90 ± 0.14 mmol/L for *L. rhamnosus* GG.

### Effects of dietary postbiotics in juvenile black rockfish

#### Growth performance, feed utilization, and body indices

**Fig. 2:**
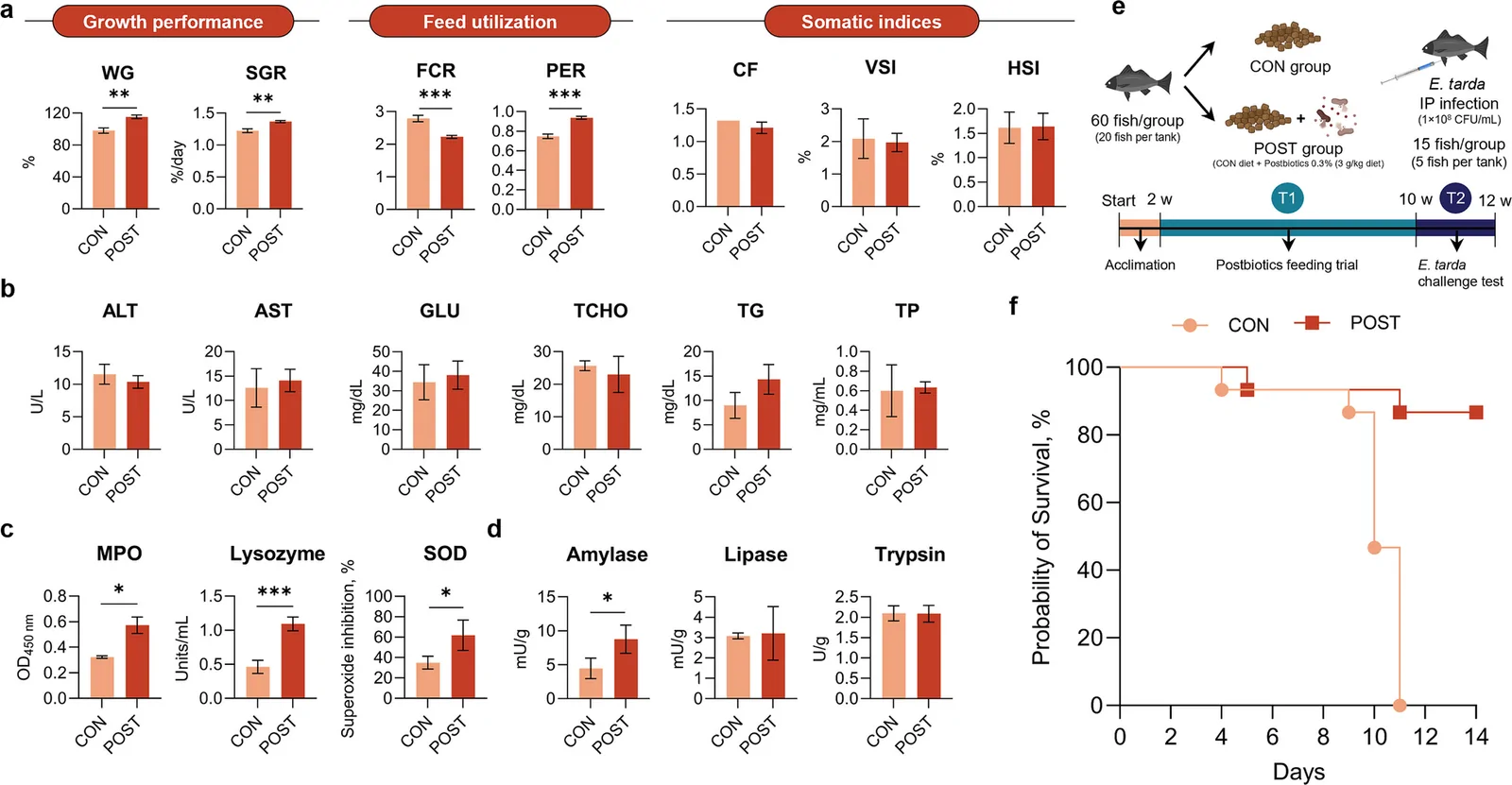
Effects of dietary postbiotic supplementation on growth performance, physiological parameters, and disease resistance in juvenile black rockfish (*Sebastes schlegelii*). The control group (CON) was fed a basal diet, whereas the POST group received a diet supplemented with postbiotics derived from *Latilactobacillus curvatus* NBL2305. **a** Growth performance, feed utilization, and somatic indices after an 8-week feeding trial, including weight gain (WG), specific growth rate (SGR), survival rate (SR), feed conversion ratio (FCR), protein efficiency ratio (PER), hepatosomatic index (HSI), viscerosomatic index (VSI), and condition factor (CF) (*n* = 15). **b** Serum biochemical parameters, including total protein (TP), triglycerides (TG), glucose (GLU), total cholesterol (TCHO), alanine aminotransferase (ALT), and aspartate aminotransferase (AST) (*n* = 5). **c** Non-specific immune parameters in serum (*n* = 5). **d** Digestive enzyme activities measured in intestinal tissue (*n* = 5). **e** Experimental design of the feeding and challenge trial, in which fish were fed the experimental diets for 8 weeks (T1) followed by a bacterial challenge test (T2) with *Edwardsiella tarda*. **f** Kaplan–Meier survival curves of juvenile rockfish following *E. tarda* challenge (*n* = 15). Data are presented as mean ± SD. Statistical significance was determined using Student’s *t*-test, with differences indicated as ^*^P < 0.05, ^**^P < 0.01, and ^***^P < 0.001. CON, control group; POST, postbiotic-supplemented group

The POST group showed significantly higher WG and SGR than the CON group (*P* = 0.002). In terms of feed utilization, the POST group exhibited a significantly lower FCR (*P* < 0.001) and a significantly higher PER (*P* < 0.001) compared with the CON group. In contrast, no significant differences (*P* > 0.05) were observed in somatic indices between the groups (Fig. 2a, Table S4).

#### Serum biochemical and non-specific immune parameters

The six serum biochemical parameters did not differ significantly (*P* > 0.05) between the CON and POST groups. However, the POST group showed significantly higher activities of the three non-specific immune parameters assessed (Myeloperoxidase (MPO), SOD, and lysozyme) compared with the CON group (Fig. 2b and c).

#### Digestive enzyme activities

The activities of three digestive enzymes (amylase, lipase, and trypsin) were evaluated in the intestinal tissues of juvenile black rockfish at the end of the feeding trial. Among these, amylase activity was significantly higher in the POST group than in the CON group (*P* = 0.044) (Fig. 2d).

#### Challenge test against *E. tarda*

The schematic design of the *E. tarda* challenge test is shown in Fig. 2e, and cumulative survival curves are presented in Fig. 2f. The first mortality in the POST group was observed on d 5 post-challenge, whereas mortality in the CON group began on d 4. Thereafter, survival in the CON group declined sharply, reaching 0.00% by d 12. In contrast, the POST group maintained a final survival rate of 86.67% at d 14. Kaplan–Meier survival analysis demonstrated a significantly higher cumulative survival rate in the POST group than in the CON group (log-rank test, χ^2^ = 20.35, df = 1, *P* < 0.001).

#### Expression of immune- and growth-related genes

**Fig. 3:**
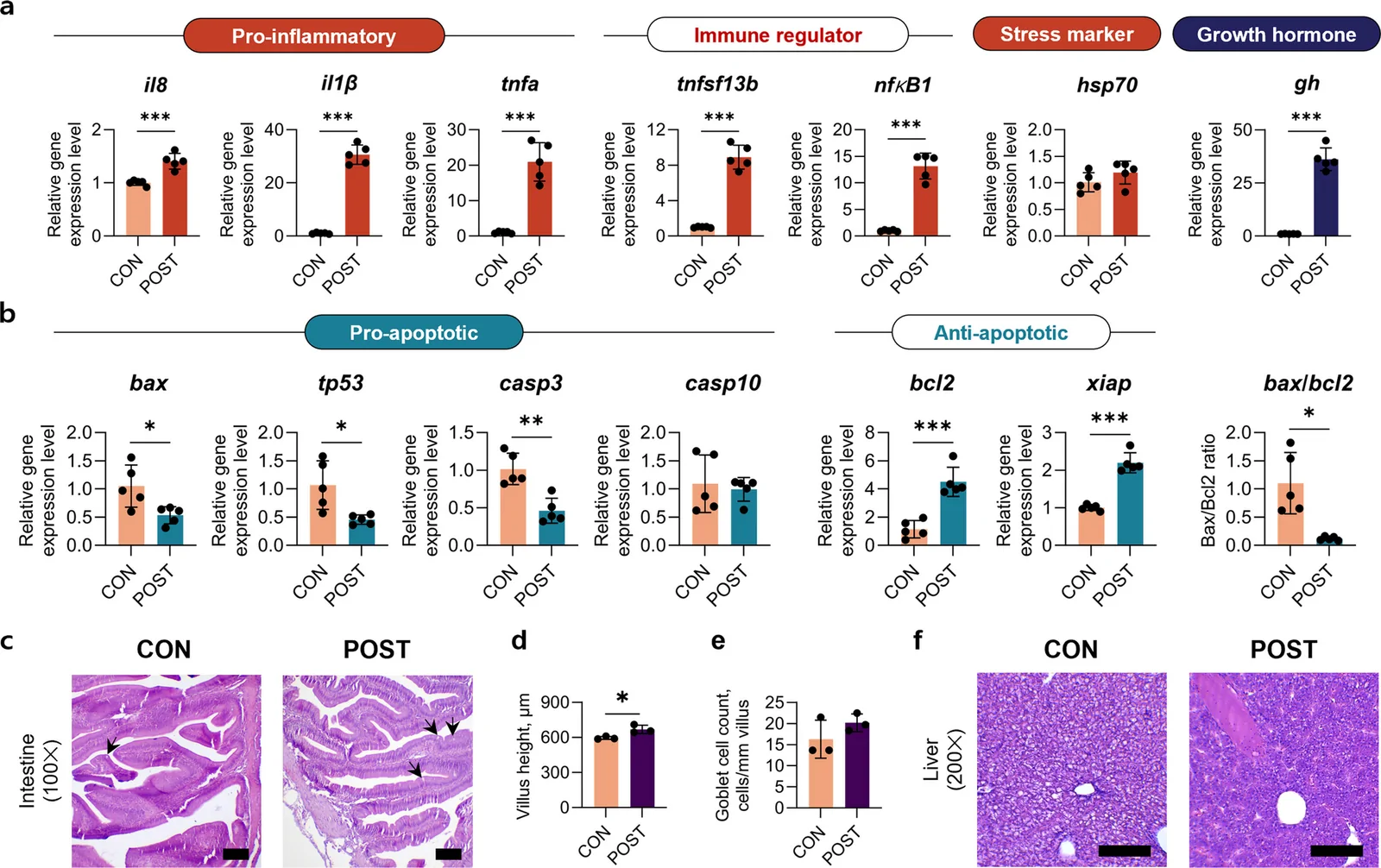
Histological changes and expression of immune-, growth-, and apoptosis-related genes in juvenile black rockfish (*Sebastes schlegelii*) following dietary supplementation with *Latilactobacillus curvatus* NBL2305-derived postbiotics. The control group is designated as CON, and the *L. curvatus* NBL2305-fed group is designated as POST. **a** Relative expression levels of immune-related genes (*il8, il1β, tnfα*, *tnfsf13b* (BAFF), *nfκB1, hsp70*) and the growth-related gene (*gh*) in intestinal tissue (*n* = 5). **b** Relative expression levels of apoptosis-related genes in liver tissue, including the pro-apoptotic genes (*bax, tp53, casp3*, and *casp10*), the anti-apoptotic genes (*bcl2* and *xiap*), and the *bax/bcl2* ratio (*n* = 5). Gene expression levels were determined by reverse transcription-quantitative polymerase chain reaction (RT-qPCR) and normalized to β-actin. **c** Representative hematoxylin and eosin (H&E) stained sections of the intestine from the CON and POST groups (*n* = 3). **d** Quantitative analysis of villus height (*n* = 3). **e** Goblet cell count per villus (*n* = 3). **f** Representative H&E-stained sections of liver tissue from the CON and POST groups (*n* = 3). Scale bars = 100 μm. Data are presented as mean ± SD. Statistical significance was determined using Student's *t*-test, with differences indicated as ^*^P < 0.05, ^**^P < 0.01, and ^***^P < 0.001

The expression of immune- and growth-related genes in the intestine is shown in Fig. 3a. In the POST group, the expression levels of the pro-inflammatory genes *il8* (*P* < 0.001), *il1β* (*P* < 0.001), and *tnfα* (*P* < 0.001) were significantly upregulated compared with those in the CON group. The immune-related genes *tnfsf13b* (B-cell activating factor, BAFF) (*P* < 0.001) and *nfkb1* (Nuclear factor kappa B, NF-κB) (*P* < 0.001) were also significantly upregulated in the POST group. In addition, the growth-related gene *gh* was significantly upregulated in the POST group (*P* < 0.001), whereas no significant difference (*P* = 0.187) was observed in *hsp70* expression between the groups. The expression of apoptosis-related genes in the liver is shown in Fig. 3b. The POST group exhibited significantly lower expression levels of the pro-apoptotic genes *bax* (*P* = 0.021), *tp53* (*P* = 0.032), and *casp3* (*P* = 0.002) compared with the CON group, while *casp10* expression did not differ significantly between the groups (*P* = 0.706). In contrast, the anti-apoptotic genes *bcl2* (*P* < 0.001) and *xiap* (*P* < 0.001) were significantly upregulated in the POST group. Accordingly, the *bax/bcl2* ratio was significantly lower in the POST group than in the CON group (*P* = 0.016).

#### Histopathological analysis

As shown in Fig. 3c, no apparent histopathological alterations were observed in the intestinal or liver tissues of either the CON or POST group. Both groups exhibited normal tissue architecture without evident lesions or structural abnormalities, indicating that dietary postbiotics supplementation did not induce adverse histological changes under the experimental conditions. Dietary supplementation with NBL2305-derived postbiotics significantly increased villus height in the POST group compared with the CON group (CON: 595.27 ± 12.73 μm; POST: 668.35 ± 35.44 μm; *P* = 0.029; Fig. 3d). Goblet cell density did not differ significantly between the CON and POST groups (CON: 16.26 ± 4.50 cells/mm villus; POST: 20.17 ± 2.11 cells/mm villus; *P* = 0.245; Fig. 3e).

#### Microbiota analysis

**Fig. 4:**
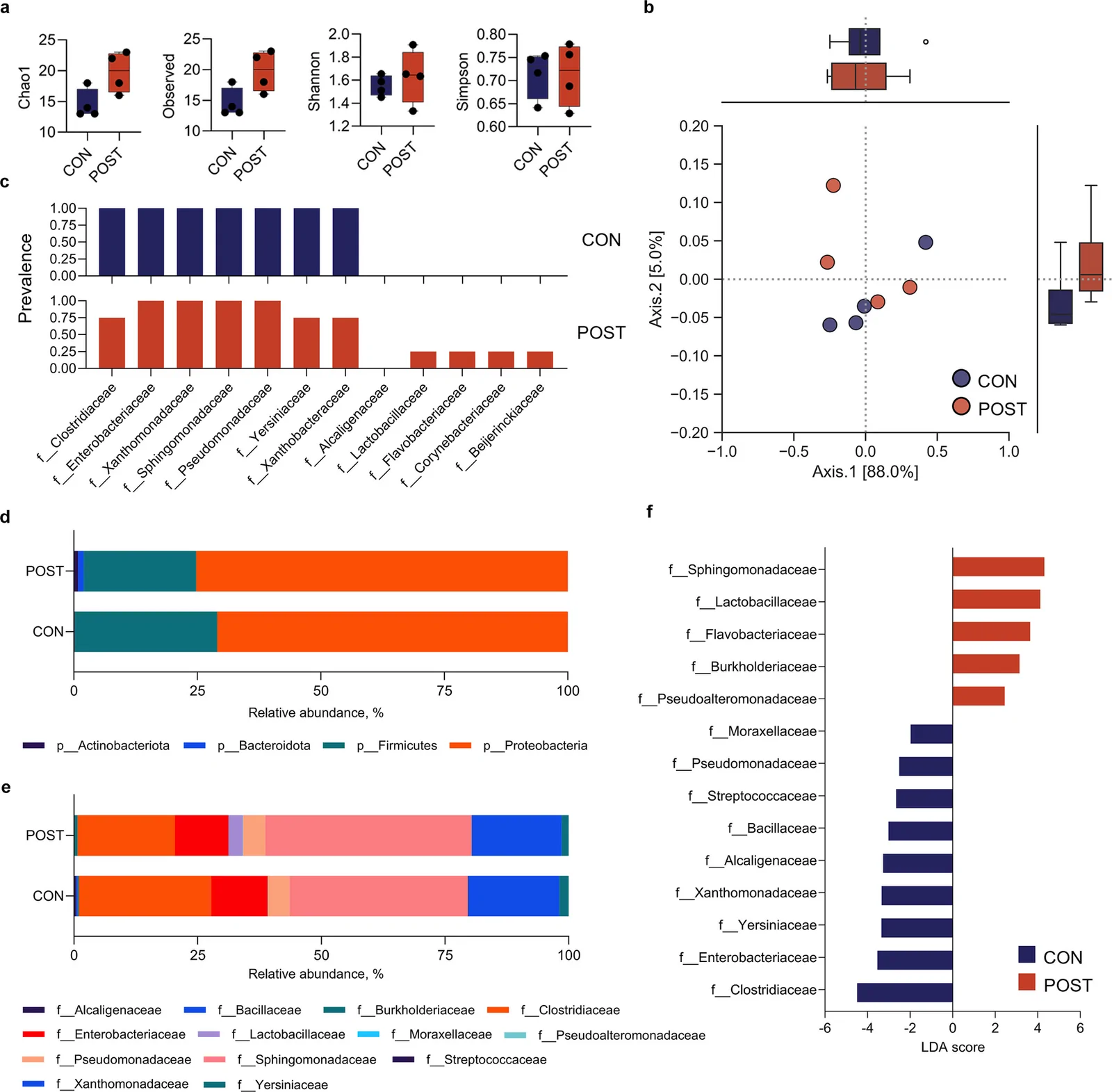
Intestinal microbiota analysis of juvenile black rockfish (*Sebastes schlegelii*) in the CON and POST groups (*n* = 4). **a** Alpha diversity indices of the intestinal microbiota, including Chao1 richness, observed features, Shannon diversity, and Simpson diversity. **b** Beta diversity of microbial community structure based on Bray–Curtis dissimilarity, visualized by principal coordinates analysis (PCoA) at the family level, with statistical significance assessed using permutational multivariate analysis of variance (PERMANOVA). **c** Core microbiome analysis showing the prevalence of shared taxa across samples. **d** and **e** Relative abundance of intestinal microbial communities at the phylum and family levels, respectively, in the CON and POST groups. **f** Differentially abundant taxa between groups identified by LEfSe, presented as linear discriminant analysis (LDA) scores. Data are presented as mean ± SD

The intestinal microbiota profiles of juvenile black rockfish in the CON and POST groups are shown in Fig. 4. Alpha diversity indices, including Chao1 richness, observed features, Shannon diversity, and Simpson diversity, did not differ significantly between the groups (*P* > 0.05; Fig. 4a). Similarly, beta diversity based on Bray–Curtis dissimilarity at the family level showed no significant difference between the CON and POST groups according to Permutational multivariate analysis of variance (PERMANOVA) (F = 0.252, *R*^2^ = 0.040, *P* = 0.779; Fig. 4b).

Core microbiome analysis revealed several taxa with high prevalence in both groups, although some family-level taxa exhibited group-specific prevalence patterns (Fig. 4c). At the phylum level, both groups were dominated by Proteobacteria, followed by Actinobacteriota, Bacteroidota, and Firmicutes (Fig. 4d). At the family level, the overall microbial community composition was largely similar between the CON and POST groups, with differences in the relative abundance of several taxa (Fig. 4e). Linear discriminant analysis effect size (LEfSe) analysis identified Lactobacillaceae as an enriched taxon in the POST group (Fig. 4f), while other differentially abundant taxa are presented in the figure. Exploratory functional prediction using PICRUSt2 identified nominal differences (raw *P* < 0.05) in predicted MetaCyc pathways between the CON and POST groups. The differentially abundant pathways were primarily associated with carbohydrate metabolism, amino acid metabolism, nucleotide/cofactor metabolism, central carbon metabolism, and nitrogen metabolism (Table S4). However, none of the predicted pathways remained significant after false discovery rate correction.

## Discussion

In this study, postbiotics derived from *Latilactobacillus curvatus* NBL2305 were characterized and evaluated for their potential as functional feed additives in juvenile black rockfish aquaculture. The initial characterization of the parental strain NBL2305 was performed to verify its safety and probiotic-related functional properties as a suitable source microorganism for postbiotic production and revealed several desirable characteristics. The strain exhibited strong tolerance to simulated gastric conditions across a wide pH range and maintained viability under salinity levels relevant to marine aquaculture, indicating good adaptation to the physiological and environmental conditions encountered by marine fish. Although survival decreased under simulated intestinal conditions at higher bile salt concentrations, partial viability was retained at 0.1% bile salts. This suggests that NBL2305 possesses moderate gastrointestinal resilience, supporting its suitability as a parental strain for postbiotic production and providing useful information regarding the biological characteristics of the producing microorganism.

NBL2305 also demonstrated a notably high adhesion capacity, significantly exceeding that of *Lactobacillus rhamnosus* GG. Adhesion is widely regarded as a key probiotic trait, as it facilitates transient interactions with the intestinal mucosa and supports host–microbe interactions [46]. Although adhesion is not strictly required for postbiotics functionality, evaluating the adhesion ability of the parental strain provides additional information regarding its probiotic-related functional properties and biological characteristics [46, 47].

In this context, the present study focused on postbiotic supplementation rather than the administration of live microorganisms. While postbiotics are defined as non-viable microbial preparations, characterization of the parental strain remains an essential step in their development because it provides complementary information regarding strain safety and functional properties [11, 48, 49]. Collectively, these findings highlight the functional potential of NBL2305 as a postbiotic-producing strain and support its applicability in aquaculture systems. In addition to functionality, safety is a critical consideration for practical application in aquaculture [11]. In the present study, NBL2305 exhibited no hemolytic activity, and all MIC values were below the cut-off thresholds established by the European Food Safety Authority [50].

Moreover, both probiotic and postbiotic forms showed no cytotoxic effects in HT-29 cells. These results support the short-term biological safety of both the strain and its derived metabolites. Importantly, the in vivo feeding trial further supported these findings. No adverse effects were observed in serum biochemical parameters, histopathological features, or somatic indices in postbiotic-fed fish. These observations are consistent with previous aquaculture studies reporting that dietary postbiotics do not induce detrimental physiological or histological changes, further supporting their safe use as feed additives [14, 51].

Postbiotics are recognized for their diverse beneficial effects, including antimicrobial and antioxidant activities [52]. In the present study, NBL2305-derived postbiotics exhibited clear inhibitory activity against six major fish pathogens, including *E. tarda*. This broad-spectrum antimicrobial effect suggests that bioactive components in the postbiotic preparation may effectively suppress key bacterial pathogens in aquaculture systems. Such properties are particularly valuable in the context of increasing efforts to reduce antibiotic use in fish farming [2]. However, the specific bioactive compounds responsible for this antibacterial activity were not identified in the present study. Therefore, the observed antimicrobial effects should be interpreted as the biological activity of the whole postbiotic preparation rather than that of any individual constituent [13, 14, 51]. Consistent with these observations, previous studies have reported that postbiotics enhance disease resistance in aquaculture species. For example, postbiotics derived from *Weissella cibaria* improved resistance to *Yersinia ruckeri* in rainbow trout [14], while heat-killed *Pediococcus pentosaceus* enhanced resistance to *Vibrio parahaemolyticus* infection in white shrimp [13]. Together, these findings further support the potential application of postbiotics for bacterial disease control in aquaculture. The in vitro antibacterial activity was further supported by the in vivo challenge test, in which the POST group exhibited a markedly higher survival rate than the CON group following *E. tarda* infection. Although mortality appeared earlier in the POST group, overall survival was substantially improved, whereas the CON group experienced rapid mortality and reached 0% survival by d 12. These results indicate that dietary NBL2305 postbiotics enhance host resistance to bacterial infection and confer biologically meaningful protection under pathogenic stress.

In addition to their antimicrobial effects, NBL2305-derived postbiotics demonstrated considerable antioxidant capacity across multiple assays. Given that oxidative stress is closely linked to immune dysfunction and tissue damage in aquaculture species [53], these antioxidant properties may help maintain physiological homeostasis and support host defense mechanisms [54, 55]. Therefore, the combined antimicrobial and antioxidant activities of NBL2305 postbiotics are likely to act synergistically in enhancing disease resistance in juvenile black rockfish.

The beneficial effects of dietary NBL2305 postbiotics were also evident in growth performance. The POST group showed significantly higher WG and SGR, along with improved feed utilization, as reflected by lower FCR and higher PER. Furthermore, amylase activity was significantly increased in the POST group, suggesting that improved carbohydrate digestion may have contributed, at least in part, to the enhanced growth performance. Although trypsin and lipase activities were not significantly affected, the increased amylase activity indicates that modulation of digestive function may represent one of several factors contributing to the observed growth response [56, 57]. The upregulation of intestinal growth hormone (GH) expression further suggests that postbiotic supplementation may influence growth-related physiological pathways. However, the observed growth enhancement is likely the result of multiple physiological responses rather than GH expression alone.

The immunological results further indicate that NBL2305 postbiotics exert pronounced immunostimulatory effects. The POST group exhibited significantly elevated activities of key non-specific immune markers, including MPO, SOD, and lysozyme. MPO contributes to microbial killing through the generation of reactive oxygen species [20], whereas SOD protects host tissues from oxidative damage by scavenging superoxide radicals [22]. Lysozyme, a critical component of innate immunity, enhances host defense by degrading bacterial cell walls [58].

Consistent with these functional responses, the POST group also showed increased intestinal expression of *il-8*, *il1β*, *tnfα*, *tnfsf13b*, and *nfkB1*, suggesting coordinated activation of immune signaling pathways. Although postbiotics are non-viable microbial preparations, their bioactive components, such as cell wall fractions and peptidoglycan, can interact with host immune cells and modulate cytokine production [17, 59]. Accordingly, the upregulation of these genes may reflect activation or priming of innate immune responses in fish [18, 19]. Among these genes, *il1β* and *tnfα* are key pro-inflammatory cytokines that initiate innate immune responses and mediate inflammatory signaling during pathogen invasion [60, 61]. *il8* functions as a chemokine that recruits immune cells, particularly neutrophils, to sites of infection, reinforcing the first line of defense [62]. *nfkB1* serves as a central transcription factor regulating a broad array of immune-related genes, including pro-inflammatory cytokines, and plays a key role in both innate and adaptive immunity [63]. In addition, *tnfsf13b*, a member of the tumor necrosis factor superfamily, is essential for B cell activation, proliferation, and survival, thereby linking innate signaling to mucosal and humoral immune responses [64]. The elevation of MPO and lysozyme activities is consistent with the upregulation of pro-inflammatory cytokines and NF-κB signaling, whereas the increased SOD activity likely reflects a compensatory antioxidant response during immune activation [21, 65–67]. Together, these findings suggest that NBL2305 postbiotics modulate innate immune responses.

**Fig. 5:**
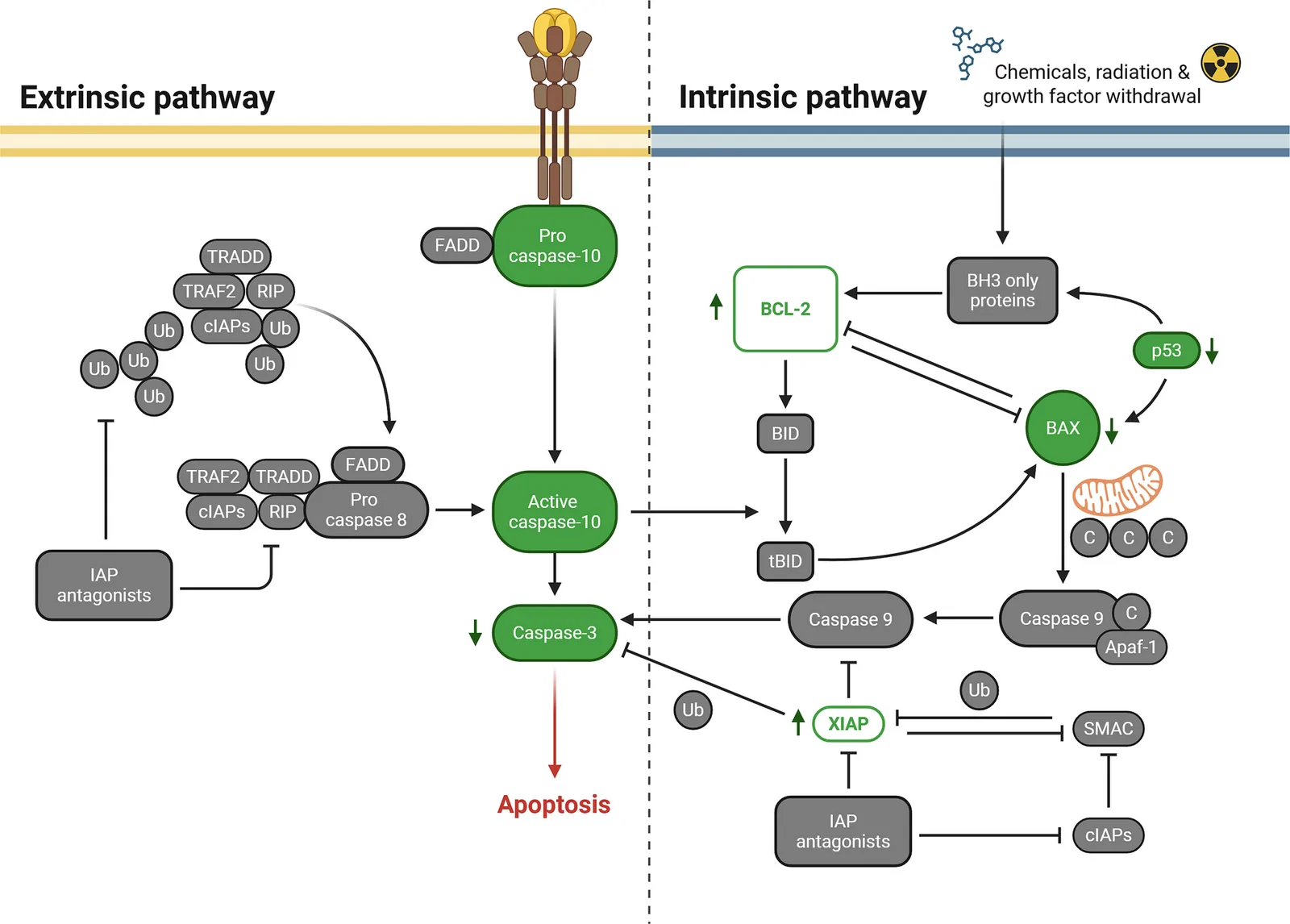
Proposed modulation of the intrinsic apoptotic signaling pathway by dietary *Latilactobacillus curvatus* NBL2305-derived postbiotics in juvenile black rockfish (*Sebastes schlegelii*). The schematic illustrates the intrinsic and extrinsic apoptosis signaling pathways, highlighting the apoptosis-related genes analyzed in the present study. Green upward (↑) and downward (↓) arrows adjacent to the highlighted genes indicate the direction of gene expression changes observed in the POST group relative to the CON group in the present study. The highlighted genes summarize the apoptosis-related gene expression changes observed in the present study and are presented within the context of the established apoptosis signaling pathways to facilitate interpretation of the findings. Created with BioRender.com

Apart from immune modulation, dietary postbiotic supplementation influenced apoptosis-related gene expression in the liver. As illustrated in Fig. 5, apoptosis is regulated through both extrinsic and intrinsic pathways, with mitochondrial signaling playing a central role in integrating stress and survival cues [23, 68]. *bax*, *tp53*, and *casp3* are key pro-apoptotic factors that promote programmed cell death in response to cellular stress, whereas *bcl2* and *xiap* act as anti-apoptotic regulators that inhibit apoptosis and support cell survival [23, 24, 67]. The balance between these opposing signals, often represented by the *bax/bcl2* ratio, is a critical determinant of cellular fate [69]. In the present study, the POST group showed downregulation of pro-apoptotic genes alongside upregulation of anti-apoptotic genes, resulting in a significantly lower *bax/bcl2* ratio. This shift suggests modulation of apoptosis-related gene expression toward an anti-apoptotic profile, indicating that NBL2305 postbiotics may contribute to the regulation of apoptosis-related pathways under physiological and stress conditions.

Notably, immune and apoptosis-related pathways are functionally interconnected. NF-κB signaling not only regulates inflammatory responses but also promotes cell survival by inducing anti-apoptotic genes such as *bcl2* and *xiap* [63, 70]. In contrast, excessive or prolonged inflammatory signaling can trigger apoptosis through pathways involving *tp53* and caspases, particularly under conditions of sustained cellular stress or immune overactivation [25, 71]. Therefore, maintaining a balance between immune activation and cell survival is essential for preserving tissue homeostasis. In the present study, the upregulation of immune-related genes in the intestine was accompanied by increased expression of anti-apoptotic genes and modulation of apoptosis-related gene expression in the liver, without any observable histopathological alterations. These findings indicate that dietary NBL2305 postbiotics were associated with modulation of innate immune responses and apoptosis-related gene expression without apparent histopathological alterations. This coordinated response may contribute to the maintenance of tissue homeostasis under physiological conditions. To the best of our knowledge, previous aquaculture studies have predominantly reported either immunomodulatory effects or histopathological safety following dietary probiotic or postbiotic supplementation [14, 26, 72]. However, studies simultaneously evaluating immune-related gene expression, hepatic apoptosis pathways, and histopathological outcomes remain limited.

Another important finding is that dietary postbiotic supplementation improved host physiological and immune parameters without disrupting intestinal microbial homeostasis. Because the CON group did not represent a disease or dysbiosis model, substantial shifts in microbial diversity or community structure were neither expected nor necessarily beneficial. Accordingly, the absence of significant differences in alpha and beta diversity between the CON and POST groups suggests that NBL2305 postbiotics preserved the overall microbial balance of the intestine. At the same time, LEfSe analysis identified Lactobacillaceae as an enriched taxon in the POST group, suggesting selective modulation of specific bacterial taxa without broadly altering the resident microbiota. This selective modulation is consistent with previous reports suggesting that postbiotics can influence the abundance of bacterial taxa, including *Lactobacillus* and *Bifidobacterium* [73]. Collectively, these findings suggest that NBL2305 postbiotics maintain intestinal microbial stability while selectively modulating specific bacterial taxa. Exploratory functional prediction further suggested nominal differences in pathways related to carbohydrate and amino acid metabolism. Although these pathways were not significant after FDR correction, they may provide preliminary insight into potential functional shifts associated with postbiotic supplementation.

An important strength of the present study is the integrated evaluation of innate immune responses, apoptosis-related gene expression, intestinal microbiota, and disease resistance within the same experimental framework. Although direct mechanistic interactions among these biological responses were not investigated, the concurrent modulation of these parameters suggests that the biological effects of NBL2305-derived postbiotics extend beyond improvements in growth performance or isolated immune markers. This integrated approach broadens current understanding of the multifunctional roles of postbiotics in aquaculture species [40].

Taken together, the present findings indicate that dietary NBL2305 postbiotics were well tolerated and functionally beneficial in juvenile black rockfish under the experimental conditions evaluated. Supplementation improved growth performance, enhanced innate immune responses, modulated immune- and apoptosis-related gene expression, exhibited antibacterial and antioxidant activities, and increased survival following *E. tarda* challenge, without inducing histopathological alterations or causing major alterations in overall intestinal microbial composition. These findings support the potential of NBL2305 postbiotics as a promising dietary strategy for improving fish health and disease resistance, with potential application as a sustainable feed additive in marine aquaculture.

Despite these promising outcomes, several limitations should be acknowledged. First, the specific bioactive components responsible for the observed antibacterial, antioxidant, and immunomodulatory effects were not identified. Second, the feeding trial was conducted using a single species and developmental stage, which may limit the generalizability of the findings to other aquaculture species or life stages. In addition, microbiota analysis was performed at the family level, restricting the taxonomic resolution of microbial changes associated with postbiotic supplementation. Moreover, functional prediction analysis was exploratory and based on 16S rRNA gene profiles rather than direct measurements of microbial metabolic activity. Furthermore, compared with mammalian systems, the immune mechanisms and intestinal microbial ecology of fish remain less well characterized, requiring additional fundamental research to better elucidate host–microbe interactions in aquaculture species. Therefore, although NBL2305 postbiotics show considerable potential as a functional feed additive for improving fish health, further studies are required to identify the active compounds, clarify the underlying mechanisms, and validate their efficacy across diverse aquaculture conditions before their broader application in diverse aquaculture production systems.

## Conclusion

In conclusion, postbiotics derived from *Latilactobacillus curvatus* NBL2305 showed considerable potential as functional dietary additives for juvenile black rockfish. Dietary supplementation improved growth performance and feed utilization, enhanced non-specific immune responses, modulated immune- and apoptosis-related gene expression, and significantly increased survival following *Edwardsiella tarda* challenge. In addition, the postbiotic preparation exhibited antibacterial and antioxidant activities. These beneficial effects were observed without detectable cytotoxicity, hemolytic activity, histopathological alterations, or major changes in overall intestinal microbial composition, supporting the biological safety of the postbiotic preparation under the experimental conditions evaluated. Collectively, these findings support the potential of NBL2305-derived postbiotics as a promising functional feed additive for improving fish health and disease resistance, with potential application as a sustainable strategy for reducing antibiotic dependence in marine aquaculture. Nevertheless, further studies are required to identify the active bioactive compounds, clarify the underlying mechanisms, and validate their efficacy across diverse aquaculture species and production conditions.

## Supplementary Information

Additional file 1: Table S1. Gene-specific primers used to quantify relative gene expression. Table S2. Composition of the basal experimental diet for rockfish. Table S3. Carbohydrate fermentation profile of *Latilactobacillus curvatus* NBL2305 determined using the API 50 CHL system. Table S4. Effects of dietary NBL2305 postbiotics on growth performance, feed utilization, and somatic indices in juvenile black rockfish. Table S5. Predicted MetaCyc pathways showing nominal differencesbetween the CON and POST groups based on PICRUSt2 functional prediction.

## Data Availability

The datasets generated and/or analysed during the current study are available from the corresponding author on reasonable request.

## Abbreviations

ABTS: 2,2′-Azino-bis(3-ethylbenzothiazoline-6-sulphonic acid)
ALT: Alanine aminotransferase
ASV: Amplicon sequence variant
AST: Aspartate aminotransferase
BAFF: B-cell activating factor
CF: Condition factor
CFU: Colony-forming unit
CON: Control group
DPPH2: 2-Diphenyl-1-picrylhydrazyl
DPBS: Dulbecco's phosphate-buffered saline
FCR: Feed conversion ratio
FRAP: Ferric reducing antioxidant power
GH: Growth hormone
GLU: Glucose
H&E: Hematoxylin and eosin
HSI: Hepatosomatic index
IL: Interleukin
LDA: Linear discriminant analysis
LEfSe: Linear discriminant analysis effect size
MIC: Minimum inhibitory concentration
MPO: Myeloperoxidase
NF-κB: Nuclear factor kappa B
PBS: Phosphate-buffered saline
PCoA: Principal coordinates analysis
PCR: Polymerase chain reaction
PER: Protein efficiency ratio
PICRUSt2: Phylogenetic Investigation of Communities by Reconstruction of Unobserved States 2
POST: Postbiotic-supplemented group
QIIME2: Quantitative Insights Into Microbial Ecology 2
RT-qPCR: Reverse transcription-quantitative polymerase chain reaction
SGI: Simulated gastrointestinal
SGR: Specific growth rate
SIJ: Simulated intestinal juice
SOD: Superoxide dismutase
TAC: Total antioxidant capacity
TCHO: Total cholesterol
TG: Triglycerides
TMB: 3,3′,5,5′-Tetramethylbenzidine
TP: Total protein
VSI: Viscerosomatic index
WG: Weight gain
