# Supplementary material for: Postbiotics enhance immune competence in black rockfish (Sebastes schlegelii): linking innate immunity, apoptosis, and resistance to bacterial infection
Source: J Anim Sci Biotechnol. 2026 Oct 4;17:186. doi: 10.1186/s40104-026-01502-y (PMC13634210; doi:10.1186/s40104-026-01502-y)
Supplement: Supplementary file 1 [file 40104_2026_1502_MOESM1_ESM.docx]

| **Table S1.** Composition of the basal experimental diet for rockfish | | | |  |  |  |
| --- | --- | --- | --- | --- | --- | --- |
| Ingredients | Percent (%) | Ingredients | Percent (%) | |  |  |
|  |  |  |  |  |  | |
| Anchovy FM | 35.00 | Soybean oil | 2.50 | |  | |
| Tuna by-product meal | 8.70 | Met 99% | 0.08 | |  | |
| Poultry by-product meal | 8.70 | Lys 99% | 0.47 | |  | |
| Corn gluten meal | 8.70 | Vitamin Mix^1^ | 1.00 | |  | |
| Wheat gluten | 1.35 | Mineral Mix^2^ | 1.00 | |  | |
| Starch | 8.30 | Vitamin C | 0.20 | |  | |
| Soybean meal | 13.00 | Choline | 0.50 | |  | |
| Wheat flour | 8.00 | Postbiotics | *Latilactobacillus curvatus* NBL2305-drived postbiotics 0.3% (3 g/kg diet) | |  | |
| Fish oil | 2.50 |  |  |  |  | |

^1^Vitamin premix (as mg kg^-1^ in diets): Ascorbic acid, 300; dl-Calcium pantothenate, 150; Choline bitate, 3,000; Inositol, 150; Menadion, 6; Niacin, 150; Pyridoxine. HCl, 15; Rivoflavin, 30; Thiamine mononitrate, 15; dl-α-Tocopherol acetate, 201; Retinyl acetate, 6; Biotin, 1.5; Folic acid, 5.4; Cobalamin, 0.06.

^2^Mineral premix (as mg kg^-1^ in diets): NaCl, 437.4; MgSO_4_⋅7H_2_O, 1,379.8; ZnSO_4_⋅7H_2_O, 226.4; Fe-Citrate, 299; MnSO_4_, 0.016; FeSO_4_, 0.0378; CuSO_4_, 0.00033; Ca(IO)_3_, 0.0006; MgO, 0.00135; NaSeO_3_, 0.00025.

| **Table S2.** Gene-specific primers used to quantify relative gene expression | | | | |
| --- | --- | --- | --- | --- |
| Gene | | Sense | Oligonucleotide sequence (5’ to 3’) | Product size (bp) |
| *β-actin* | Βeta-actin | F | ATGAAGGCTATGCTCTGCCC | 136 |
|  |  | R | TCACGCACGATTTCCCTCTC |  |
| *il1β* | Interleukin-1 beta | F | ACTGCACTACCAATGTCTGCT | 136 |
|  |  | R | CAATGGTGCCACCTTCTGGT |  |
| *il8* | Interleukin-8 | F | AAGAGAAGCCTGGGAGTGGA | 104 |
|  |  | R | TGGGAGTTGGCAGGAATCAG |  |
| *hsp70* | Heat shock protein 70 | F | GCACCACCTACTCCTGTGTC | 111 |
|  |  | R | TCTCTGTGTCAGTGAACGCC |  |
| *tnfα* | Tumor necrosis factor-alpha | F | TCGTGTTCACACTCTGCCTC | 130 |
|  |  | R | TTTCCGCCTGAGATTTGCCT |  |
| *nfkB1* | Nuclear factor kappa B | F | GTGTTGCTGCTGTTCTGGTG | 147 |
|  |  | R | GATGAAGGCTCAGCTCCCTG |  |
| *tnfsf13b* | B cell activating factor | F | ATGGACAGCACCTTTGCGAT | 143 |
|  |  | R | CCTCCTGTGTAGCAGGTGTT |  |
| *gh* | Growth hormone | F | CGCTTTTCCACTGTGAAGCC | 140 |
|  |  | R | GCACAGAGACACCGAACAGA |  |
| *xiap* | X-linked inhibitor of apoptosis protein | F | GAAGGCCATAGGGATGGGTCT | 148 |
|  |  | R | TGTCTTGGCAGCATCACTGTC |  |
| *bcl2* | B cell lymphoma 2 | F | TATCGGAGACCAGGAACACC | 129 |
|  |  | R | TCAGGCATAAAGCACACAGC |  |
| *bax* | Bcl2 associated X protein | F | CAGTCGGTG AGGGAGATCAT | 109 |
|  |  | R | CCGCTAATTCCGAGAAACTG |  |
| *tp53* | Tumor protein 53 | F | AGTGCTGAGGAGGAGGACAA | 141 |
|  |  | R | TTGCTTCACAGACACCTTGC |  |
| *casp3* | Cysteinyl aspartate-specific protease-3 | F | GTGTTTGGGAAGTTGGGCTA | 103 |
|  |  | R | AGCAGCTGTGATCGTCCTTT |  |
| *casp10* | Cysteinyl aspartate-specific protease-10 | F | TGTCTGGTGTGTTGCGTTCT | 118 |
|  |  | R | AAGAGGCACACTTCACTCCG |  |

| **Table S3.** Carbohydrate fermentation profile of *Latilactobacillus curvatus* NBL2305 determined using the API 50 CHL system | | | | | | | | | | | | | | | | | | | | | | | | | |
| --- | --- | --- | --- | --- | --- | --- | --- | --- | --- | --- | --- | --- | --- | --- | --- | --- | --- | --- | --- | --- | --- | --- | --- | --- | --- |
| Test  number | 0 | 1 | 2 | 3 | 4 | 5 | 6 | 7 | 8 | 9 | 10 | 11 | 12 | 13 | 14 | 15 | 16 | 17 | 18 | 19 | 20 | 21 | 22 | 23 | 24 |
| Test | Control | Glycerol | Erythritol | D-arabinose | L-arabinose | Ribose | D-xylose | L-xylose | Adonitol | β Methyl-D-Xyloside | Galactose | Glucose | Fructose | Mannose | Sorbose | Rhamnose | Dulcitol | Inositol | Mannitol | Sorbitol | Methyl-D-mannoside | Methyl-D-glucoside | N-Acetyl-Glucosamine | Amygdalin | Arbutin |
| NBL2305 | - |  |  | - | - | - | - | - | - | - | - | - | **+** | **+** | **+** | **+** | - | - | - | - | - | - | - | - | **+** |
| Test  number | 25 | 26 | 27 | 28 | 29 | 30 | 31 | 32 | 33 | 34 | 35 | 36 | 37 | 38 | 39 | 40 | 41 | 42 | 43 | 44 | 45 | 46 | 47 | 48 | 49 |
| Test | Esculin | Salicin | Cellobiose | Maltose | Lactose | Melibiose | Sucrose | Trehalose | Inulin | Melezitose | Raffinose | Starch | Glycogen | Xylitol | β-Gentiobiose | D-truanose | D-lyxose | D-tagatose | D-fucose | L-fucose | D-arabitol | L-arabitol | Glyconate | 2-Keto-Gluconate | 5-Keto-Gluconate |
| NBL2305 | - | - | - | + | - | - | **+** | - | - | - | - | - | - | - | - | - | - | - | - | - | - | - | - | - | - |

| **Table S4.** Effects of dietary NBL2305 postbiotics on growth performance, feed utilization, and somatic indices in juvenile black rockfish | | | |
| --- | --- | --- | --- |
| Parameter | CON | POST | *P*-value |
| Weight gain (WG, %) | 98.10±3.30 | 115.24±2.18 | 0.002 |
| Specific growth rate (SGR, %/day) | 1.22±0.33 | 1.37±0.02 | 0.002 |
| Feed conversion ratio (FCR) | 2.79±0.10 | 2.22±0.04 | <0.001 |
| Protein efficiency ratio (PER) | 0.75±0.03 | 0.94±0.02 | <0.001 |
| Condition factor (CF) | 1.32±0.00 | 1.21±0.09 | 0.160 |
| Viscerosomatic index (VSI, %) | 2.09±0.60 | 1.97±0.29 | 0.778 |
| Hepatosomatic index (HIS, %) | 1.61±0.32 | 1.64±0.27 | 0.918 |
| Values are presented as mean ± SD (n = 3). Statistical significance was determined using Student's t-test. | | | |

| **Table S5.** Predicted MetaCyc pathways showing nominal differences (raw *P* < 0.05) between the CON and POST groups based on PICRUSt2 functional prediction | | | | | | |
| --- | --- | --- | --- | --- | --- | --- |
| **Pathway ID** | **Pathway name** | **CON** | **POST** | **Fold change** | **P-value** | **FDR** |
| **Cell envelope biosynthesis** | | | | | | |
| PWY-7674 | CMP-8-amino-3,8-dideoxy-D-manno-octulosonate biosynthesis | 0.00 | 2818.13 | Detected only in POST | 0.02 | 0.55 |
| **Carbohydrate metabolism** | | | | | | |
| DARABCATK12-PWY | D-arabinose degradation II | 256.08 | 22.42 | 0.09 | 0.03 | 0.55 |
| GLUCOSE1PMETAB-PWY | Glucose and glucose-1-phosphate degradation | 85501.49 | 56931.09 | 0.67 | 0.03 | 0.55 |
| KETOGLUCONMET-PWY | Ketogluconate metabolism | 5065.27 | 0.00 | 0.00 | 0.02 | 0.55 |
| P441-PWY | Superpathway of *N*-acetylneuraminate degradation | 5583.88 | 59.09 | 0.01 | 0.03 | 0.55 |
| PWY-6531 | Mannitol cycle | 29131.83 | 17746.42 | 0.61 | 0.03 | 0.55 |
| **Amino acid metabolism** | | | | | | |
| ASPASN-PWY | Superpathway of *L*-aspartate and *L*-asparagine biosynthesis | 17950.76 | 11482.35 | 0.64 | 0.03 | 0.55 |
| PWY-8011 | *L*-serine biosynthesis II | 27515.23 | 19350.91 | 0.70 | 0.03 | 0.55 |
| **Nucleotide / Cofactor metabolism** | | | | | | |
| PWY-6609 | Adenine and adenosine salvage III | 59701.40 | 39017.26 | 0.65 | 0.03 | 0.55 |
| PYRIDNUCSAL-PWY | NAD salvage pathway I (PNC VI cycle) | 45751.99 | 28787.84 | 0.63 | 0.03 | 0.55 |
| **Central carbon metabolism** | | | | | | |
| P23-PWY | Reductive TCA cycle I | 71828.01 | 48150.24 | 0.67 | 0.03 | 0.55 |
| **Nitrogen metabolism** | | | | | | |
| PWY-6748 | Nitrate reduction VII | 1484.91 | 84.15 | 0.06 | 0.03 | 0.55 |
